# Ultrafiltration Patterns during Automated Peritoneal Dialysis: Findings and Insights to Peritoneal Physiology

**DOI:** 10.34067/KID.0000000000000506

**Published:** 2024-07-08

**Authors:** Osama El Shamy, Nicole Wyatt, Sagar Patel, Naief Abudaff, Robert Greevy, Andrew Guide, Ankur D. Shah, Juan Pablo Arroyo, Thomas A. Golper

**Affiliations:** 1 Division of Renal Diseases and Hypertension, George Washington University, Washington, DC; 2 Division of Nephrology and Hypertension, Vanderbilt University Medical Center, Nashville, Tennessee; 3 Renal Associates LLC, Columbus, Georgia; 4 Arizona Kidney Disease and Hypertension Center, Phoenix, Arizona; 5 Department of Biostatistics, Vanderbilt University Medical Center, Nashville, Tennessee; 6 Division of Kidney Disease and Hypertension, Warren Alpert Medical School of Brown University Providence, Providence, Rhode Island; 7 Division of Nephrology, Robert Larner College of Medicine, University of Vermont, Burlington, Vermont

**Keywords:** chronic dialysis, peritoneal dialysis, peritoneal membrane, ultrafiltration

## Abstract

**Key Points:**

- There is a consistent increase in ultrafiltration volumes achieved per cycle over the course of an automated peritoneal dialysis treatment session.
- A better understanding of intercycle ultrafiltration trends may inform prescription interventions that can improve patient retention.
- Surface area recruitment, mesenteric elasticity, and cumulative glucose concentration in the interstitium are possible explanations for our findings.

**Background:**

With the growing use of automated peritoneal dialysis (APD), it is important to improve our knowledge of the clinical patterns and physiology of APD treatment sessions. The ultrafiltration (UF) achieved during each cycle of an APD treatment is assumed to be relatively linear if the delivered prescription is the same. We set out to determine whether that is indeed the case.

**Methods:**

This is a single-center, cross-sectional study of patients on prevalent peritoneal dialysis (PD). All adult patients on APD (older than 18 years) who had been on PD for ≥3 months and ≥3 months on APD were included. Patients on continuous ambulatory PD or those with peritonitis within 3 months of the consent date were excluded. Individual treatment data from seven consecutive APD treatment sessions with consistent dialysate composition for each cycler exchange were collected for each patient.

**Results:**

Thirty-nine patients met the inclusion criteria and were enrolled. The probability of yielding a positive UF was 48.9% for cycle 1, rising to 90.5% by cycle 6. Adjusting for average dextrose concentration, dwell time, fill volume, solute transfer rate, and number of cycles, we observed that cycles 2–6 achieved progressively higher UF volumes than cycle 1 (*P* < 0.001). The first and last cycles demonstrated significantly different cycle UF volumes compared with a middle cycle (−230 and 277 ml, respectively, *P* < 0.001).

**Conclusions:**

We observed a consistent increase in UF volumes achieved per cycle over the course of an APD treatment session with numerous clinical and physiologic implications. This provides the foundation for future studies investigating peritoneal intercycle variations and membrane physiology.

## Visual Abstract

**Figure:**
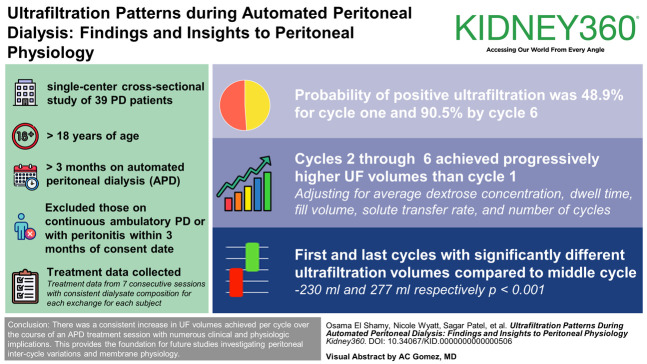

## Introduction

Peritoneal dialysis (PD) modality survival is contingent on minimizing risk factors of modality discontinuation, such as inadequate ultrafiltration (UF) and peritoneal membrane function. In many parts of the world, and especially in the United States, automated PD (APD) has become the most prevalent form of PD.^1^ Our understanding of PD physiology has been predominantly based on observations of either extended dwells or continuous ambulatory PD–based regimens.^2–6^ Minimizing membrane exposure to dextrose and preserving membrane integrity in patients on APD will require a better understanding of intercycle outcomes during APD treatments.

Solute and fluid removal are the key mandates of dialysis. In APD, independent of diurnal dwells, this is manipulated by adjusting dialysate dextrose concentration, duration of the cycler session, fill volume per cycle, and dwell time per cycle. Cardiovascular death remains the leading cause of death in patients on PD.^7,8^ Therefore, understanding the UF profile and prescription management of each patient is key to improving cardiovascular outcomes. Daily PD UF is often simply totaled. Its constituents are UF volumes achieved during APD cycles and diurnal exchange(s). Intracycle UF volumes achieved during an APD treatment vary depending on the cycler's dialysate delivery mechanism. This becomes especially significant in circumstances where patients are using two different dextrose concentration solutions during their treatments (*e.g*., 1.5% and 2.5% dextrose solutions). Some cyclers have one warmer, so the first APD cycle exchange is exclusively delivered from the PD solution bag that is placed on the warmer. Subsequently, the cycler extracts a volume of dialysate from the other bag to refill the bag that is on the warmer—*de facto* resulting in a slow shift over the course of the treatment to PD dextrose solutions infused that are almost exclusively from the subsequent bags. Other cyclers have two warmers and extract equal volumes of dialysate from all bags to infuse during each APD cycle.

With the growing usage of nocturnal APD in the United States, now constituting 86% of patients on incident PD,^1^ it is imperative that we understand the clinical patterns, outcomes, and physiology of APD patient treatment cycles. Given the importance of UF volumes achieved in APD for both fluid removal and modality survival, we examined the UF patterns occurring during each cycle of seven APD sessions. Understanding this pattern of interdwell variability in UF will improve future prescriptions and shed important insight into the physiology of osmotic-induced UF.

## Methods

This is a single-center, cross-sectional study of patients on prevalent PD. All adult patients on APD (older than 18 years) who have been on PD for ≥3 months and ≥3 months on APD were included. Patients who were on continuous ambulatory PD or had an episode of peritonitis within 3 months of the consent date were excluded.

Patients were consented between March and September 2023. Data were collected during the same month of consent. Study personnel were present in the home dialysis clinic during working hours and obtained verbal permission from the providers to approach the patients at the conclusion of their regular monthly visit. At the time of consent, patients were asked to confirm the precise PD dialysate solutions they were using.

### Data Acquisition

Two platforms were used for data acquisition: (*1*) Epic, the electronic medical record system, and (*2*) Baxter's online Sharesource. All patients were using Baxter's Amia cycler, except for one who used Baxter's HomeChoice Claria. All the patients in the study used the Baxter Dianeal and Extraneal PD solutions.

Age, sex, race, height, weight, body mass index, dialysis vintage, serum albumin concentrations, diabetes diagnosis, data collection month's dialysis kinetics (PD Kt/V_urea_ and residual kidney function Kt/V_urea_), and peritoneal equilibration test results within 12 months of data collection were all extracted from the electronic medical record. Conforming to the most recent International Society of Peritoneal Dialysis recommendations, solute *transport* is now referred to as solute *transfer*.^9^

Data from seven consecutive PD treatment days with consistent PD prescriptions (each patient was using their prescribed dialysis treatment with no changes in their prescription) were collected for each patient. The dextrose solutions used were noted at the time of patient consent and confirmed with patients 7 days after consent. Individual treatment data extracted from Sharesource included total treatment time, number of cycles per treatment, fill volumes per cycle, fill times, dwell times, drain times, UF achieved per APD cycle, net UF volume per day, night therapy UF volume per day (APD cycler session), last fill volume, initial drain volume, weights before and after PD treatment, as well as BP measurements before and after PD treatment. All the patients in the study with a last fill had an extended dwell that lasted until the initiation of their subsequent APD cycler treatment. All patients had their APD cycler temperatures set at the manufacturer's default of 36°C.

### Statistical Analyses

The primary outcome was total cycle-specific UF over the course of the fill, dwell, and drain times within each cycle. Secondary outcomes included night therapy (APD cycler session) UF and total daily UF.

Initially, various univariate analyses were conducted. Within each study day, Spearman's correlations were calculated between the patients' weight change from the start and end of the treatment with the total UF, night therapy (APD cycler session) UF, and change in BP. Additional correlations were calculated between total and night therapy UF with the average dextrose concentration of the solutions used during an APD cycler session (1.5%, 2.0%, 2.5%, 3.37%, and 4.25%). Total and night therapy UF were compared across the four transfer status groups and between those with and without a last fill (dry day).

An intercycle analysis examined the night therapy cycle-specific UF output for up to six cycles. Box plots were made comparing the cycle UF for each patient within each day to detect noticeable patterns. A separate bar chart was made to determine the probabilities of each cycle UF having a positive yield. Spearman's correlations were performed to assess the strength of the relationship between the prescribed time and the actual recorded dwell times.

The cycle times and UF may vary between patients and days, making it challenging to directly compare the UF rates between cycles. To account for this, a nonparametric analysis was conducted to compare the cycle UF by ranking them within each person-day. The UF of each cycle was compared, and the highest UF was assigned a rank of 1 and second highest a rank of 2 and continued throughout the remainder of the cycles. The results of this ranking were visualized in bar charts where the proportion of each cycle falling into each available ranking was plotted. Because potential first and last cycle effects could be hidden because of each patient having four to six cycles, three separate plots were made containing only those with either four, five, or six cycles.

A major point of emphasis was to examine associations between the cycle position and its UF volume. We conducted a longitudinal analysis using generalized estimating equations. The outcome of these models was the cycle UF, and the variable of interest was the cycle number; these models were also adjusted for potential confounders: average dextrose concentration, solute transfer rate, fill volume, and dwell time. An independent correlation structure was chosen for each of the generalized estimating equation models used. For reference groups, we used the 2% dextrose concentration, high solute transfer rate, and the first cycle as the references.

To check whether the continuous variables of dwell time and fill volume may have nonlinear associations with the cycle UF, we made a separate model with polynomial terms of degree 6 for both covariates to see if the nonlinear modeling yielded a significant association with cycle UF. We recategorized the cycle variable into a first, middle, or last cycle (reference: first cycle) to check for first and last cycle effects. Similar to Stachowska-Pietka *et al.*,^10^ we re-created a model for those with and without a last fill to ascertain whether the last fill population (carrying a dwell at least partly during the day) may have a different association between cycle position and cycle UF. A sensitivity analysis was performed using an exchangeable and autoregressive correlation structure to compare the consistency of conclusions; all models yielded similar results. All statistical analyses were conducted using R 4.2.3.

This study was approved by the Vanderbilt University Institutional Review Board (IRB-222074).

## Results

Thirty-nine patients met the inclusion criteria and were enrolled (273 APD sessions). Their demographic characteristics are summarized in Table 1. The majority had high average and low average solute transfer rates. Most of the patients (71.8%) did not have a tidal PD prescription, and the number of nocturnal PD cycles prescribed varied from four to six, with 64.1% having a last fill. Most of the nocturnal PD cycles prescribed were either 2.5% dextrose (56.4%) or a combination of 1.5% and 2.5% dextrose (28.2%).

**Table 1:** Basic summary statistics of patients' demographic and clinical data

Variable	Outcome (*N*=39)
Age, yr^a^	53.6 (15.4)
**Sex, *n* (%)**	
Female	15 (38.5)
Male	24 (61.5)
**Race, *n* (%)**	
Asian	1 (2.6)
Black	12 (30.8)
Hispanic	6 (15.4)
Middle Eastern/North African	1 (2.6)
White	19 (48.7)
Height (cm)^a^	171.2 (10.4)
Weight (kg)	83.1 (20.4)
Body mass index (kg/m^2^)^a^	28.3 (6.0)
Albumin (g/dl)^a^	3.9 (0.3)
Diabetic	17 (43.6)
On insulin	14 (35.9)
**Transfer status, *n* (%)**	
High	5 (12.8)
High average	13 (33.3)
Low average	17 (43.6)
Low	2 (5.1)
Unavailable	2 (5.1)
**Tidal PD, *n* (%)**	
No	28 (71.8)
Yes (85%)	1 (2.6)
Yes (90%)	2 (5.1)
Yes (95%)	8 (20.5)
**No. of PD cycles per treatment, *n* (%)**	
4	14 (35.9)
5	16 (41.0)
6	9 (23.1)
**Average prescribed dextrose concentration (%), *n* (%)**	
1.5	3 (7.7)
2.0 (mix 1.5% and 2.5%)	11 (28.2)
2.5	22 (56.4)
3.4 (mix 2.5% and 4.25%)	3 (7.7)
**Last fill solution, *n* (%)**	
1.5%	3 (7.7)
2.5%	2 (5.1)
Icodextrin	20 (51.3)
None	14 (35.9)
**Residual kidney function, *n* (%)**	
No	11 (28.2)
Yes	27 (69.2)
Unavailable	1 (2.6)
**Dialysis adequacy (Kt/V)^a^, *n* (%)**	
PD	1.43 (0.4)
Residual kidney function	0.46 (0.4)
Total	1.89 (0.3)
**APD cycler, *n* (%)**	
Amia	38 (97.4)
HomeChoice pro	1 (2.6)
**Drain time**	Mean (SD)
Cycle 1	17.0 (4.9)
Cycle 2	17.9 (5.5)
Cycle 3	18.0 (6.0)
Cycle 4	18.7 (5.9)
Cycle 5	22.6 (9.4)
Cycle 6	27.3 (23.4)

APD, automated peritoneal dialysis; PD, peritoneal dialysis.

a Expressed as mean (SD).

### Univariate Analysis

There was a moderate Spearman's correlation between patients' change in weight and both total UF (0.375; 95% confidence interval [CI], 0.25 to 0.49) and night therapy UF (0.342; 95% CI, 0.21 to 0.46). There was no correlation between change in weight and BP (Table 2). There was no statistically significant difference between those on tidal PD versus nontidal PD in either night therapy UF (987 versus 952 ml) or total UF (1,013 versus 1,024 ml).

**Table 2:** Spearman's correlations between change in weight per treatment and total ultrafiltration, night therapy ultrafiltration, and change in systolic, diastolic, and pulse pressures

Variable 1	Variable 2	Correlation (95% CI)
Change in weight^a^	Total UF	0.375 (0.25 to 0.49)
	Night therapy UF	0.342 (0.21 to 0.46)
	Change in systolic BP^b^	0.032 (−0.11 to 0.17)
	Change in diastolic BP^c^	0.063 (−0.07 to 0.20)
	Change in pulse pressure^d^	0.032 (−0.11 to 0.17)

CI, confidence interval; UF, ultrafiltration.

a Pretreatment minus post-treatment weight.

b Pretreatment minus post-treatment systolic BP.

c Pretreatment minus post-treatment diastolic BP.

d Pretreatment minus post-treatment pulse pressure.

There was a moderate positive Spearman's correlation between average dextrose concentration and total UF (0.410; 95% CI, 0.30 to 0.51) and night therapy UF (0.392; 95% CI, 0.28 to 0.49). There was a clear increase in the median total UF and night therapy UF achieved as the PD solution dextrose concentration increased (Supplemental Table 1).

There was no clear correlation between solute transfer status and either total or night therapy UF (Supplemental Table 1).

### Intercycle Analysis

**Figure 1:**
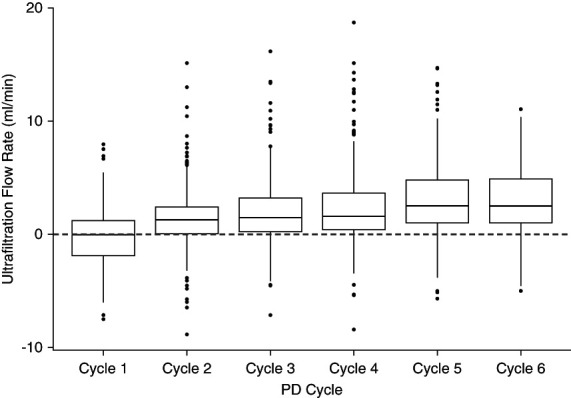
**UFRs across all PD cycle treatments.** PD, peritoneal dialysis; UF, ultrafiltration; UFR, UF flow rate.

**Figure 2:**
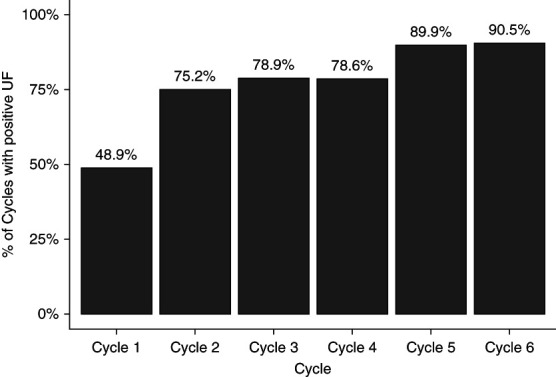
Probability that a cycle has a positive cycle UF value.

UF flow rates (UFRs) across each cycle's time (the sum of fill, dwell, and drain times) for each patient demonstrated a pattern of overall increase in UFR as the cycles progressed. The median UFR for cycle 1 was −0.1 ml/min (IQR, −1.9 to 1.2 ml/min), increasing to 1.5 ml/min by cycle 4 and as high as 2.5 ml/min in cycle 6 (Figure 1 and Table 3). The probability for a cycle to yield positive UF was as low as 48.9% for cycle 1, subsequently rising to as high as 90.5% by cycle 6 (Figure 2).

**Table 3:** Ultrafiltration flow rates per cycle

Ultrafiltration Rate	Cycle 1	Cycle 2	Cycle 3	Cycle 4	Cycle 5	Cycle 6
UFR (ml/min)^a^	−0.1 (−1.9 to 1.2)	1.2 (0.0–2.4)	1.5 (0.2–3.2)	1.5 (0.4–3.6)	2.4 (1.0–4.8)	2.5 (1.0–4.9)

UFR, ultrafiltration flow rate.

a Expressed as median (interquartile range).

**Figure 3:**
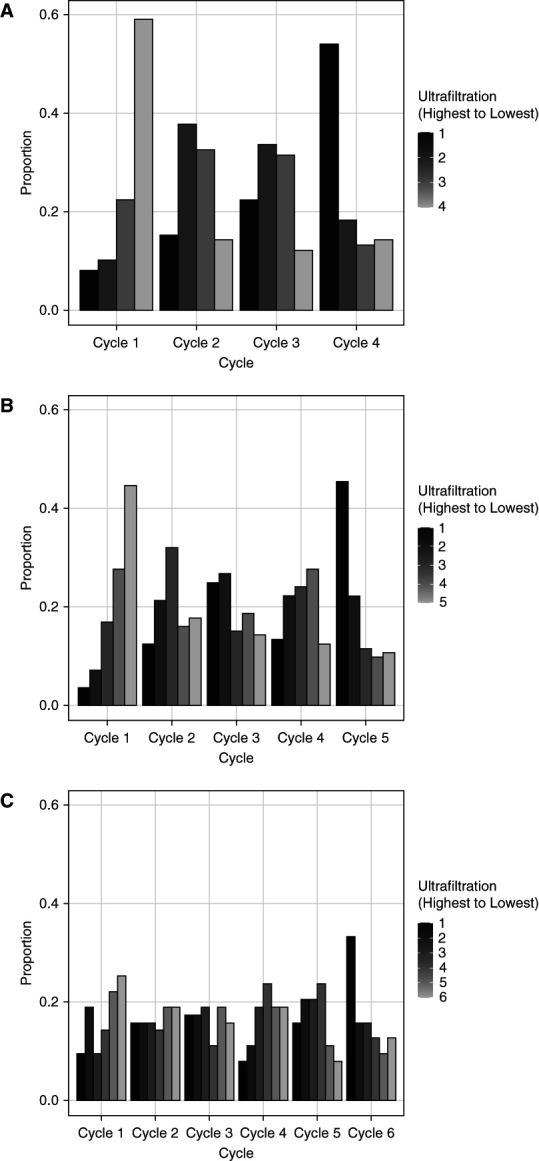
**Ranking each cycle by the proportion of the total UF achieved during a treatment session.** Each cycle is ranked from highest (darkest bar) to lowest (lightest bar) contribution to UF within each person-day treatment session. Daily UF proportion rankings across each cycle for patients who were prescribed four cycles (A), five cycles (B), and six cycles (C) per PD treatment.

In patients who were prescribed four cycles, cycle 1 provided the lowest cycler session UF proportions, and as the cycles progressed, the proportion of UF achieved per cycle increased (Figure 3A). The same pattern was seen for patients who were prescribed five or six cycles (Figure 3, B and C).

### Multivariate Analysis

Longitudinal analysis of cycle UF (Table 4), adjusting for average dextrose concentration, dwell time, tidal PD status, fill volume, solute transfer status, and number of cycles, demonstrated that—compared with cycle 1 in patients with a high solute transfer rate using 2% dextrose—cycles 2–6 had significantly higher UF volumes (*P* < 0.001).

**Table 4:** Predicting cycle ultrafiltration using the generalized estimating equation longitudinal model analysis, adjusting for average dextrose concentration, dwell time, tidal peritoneal dialysis status, fill volume, transfer status, and cycle number^a^

Variable	Estimate (SEM)	*P* Value
**Average dextrose concentration, %**		
1.5	32.39 (64.01)	0.613
2.5	77.20 (27.19)	0.005
3.38	124.51 (24.41)	<0.001
Dwell time	−0.09 (0.86)	0.917
Fill volume	31.45 (31.23)	0.314
**Transfer status**		
High average	22.78 (24.38)	0.350
Low average	136.54 (32.49)	<0.001
Low	66.82 (63.32)	0.291
Unavailable	12.22 (45.33)	0.788
**Cycle number**		
2	197.74 (40.41)	<0.001
3	258.31 (44.44)	<0.001
4	322.28 (76.01)	<0.001
5	420.50 (76.31)	<0.001
6	417.47 (93.55)	<0.001

a Reference categories were average dextrose concentration 2.0, transfer status: high, and cycle 1.

A nonlinear dwell time analysis (Table 5) was then undertaken by categorizing cycles as first, middle, or last, to factor for a potential last cycle effect. The first and last cycles demonstrated significantly different cycle UF volumes (−230 and 277 ml, respectively, *P* < 0.001).

**Table 5:** Predicting cycle ultrafiltration using the generalized estimating equation nonlinear model analysis and recategorizing cycles into first, middle, and last

Variable	Estimate (SEM)	*P* Value
**Average dextrose concentration**		
1.5%	−0.55 (57.03)	0.992
2.5%	58.98 (28.53)	0.039
3.38%	100.53 (47.41)	0.034
Dwell time	−36.40 (45.20)	0.421
Fill volume	−25.20 (39.90)	0.528
**Transfer status**		
High average	−10.61 (27.50)	0.700
Low average	82.24 (24.91)	<0.001
Low	72.13 (32.32)	0.003
Unavailable	4.58 (20.93)	0.827
**Cycle**		
First	−230.83 (34.48)	<0.001
Last	277.34 (65.43)	<0.001

Adjustments were made for average dextrose concentration, dwell time, tidal peritoneal dialysis status, fill volume, transfer status,^a^ and cycle number.

a Reference categories were average dextrose concentration 2.0, transfer status: high, and middle cycle.

### Internal Validation Observations

The median total UF achieved in patients with a last fill was slightly higher than those without a last fill, but the night therapy UF was similar in both groups (Supplemental Table 2). The night therapy UF comprised the majority of the total UF (Supplemental Table 2).

There was very strong Spearman's correlation between prescribed cycler session length and the actual cycler session duration time (Supplemental Table 3). There was also a consistent correlation between the length of each cycler treatment session and each cycle's dwell time (Supplemental Table 3), demonstrating that the cycle dwell times did not differ significantly from one cycle to the next.

Statistically significantly higher cycle UF volumes were seen with dextrose concentrations of 2.5% and 3.38% and in patients with low average solute transfer rates (Tables 4 and 5). Additional analysis stratified by last fill yielded similar results, although the presence of a last fill resulted in less variability between first and last cycle UF volumes (−171 versus −334 ml and 257 versus 316 ml) (Supplemental Table 4). A likelihood ratio test comparing models with and without a covariate interacting last fill status with the cycle position indicated that there is a significant effect of the presence/absence of a last fill on the cycle position's effect on the UF (*P* = 0.004). Similar results were seen with removing dwell time and fill volume from the adjustment model (Supplemental Table 5).

## Discussion

Using analyses adjusting for PD solution dextrose concentration, dwell time, fill volume, and peritoneal solute transfer status, as well as linear, nonlinear, and devoid analyses of dwell time and fill volume, we demonstrated consistent increase in UF volumes as the APD treatment progressed. Individual patients' session duration, number of cycles, fill volume, solution composition, and temperature did not change over the course of the week of data collection. All but one patient used Baxter's Amia APD cycler, which is programmed to pull equal volumes of dialysate from each connected PD dialysate bag. The APD treatments were performed at night, so another presumption is that the major body position was supine and intraperitoneal hydrostatic pressures were relatively consistent. As the APD treatment progressed, the UF per cycle increased, as did the likelihood of net positive UF. Regardless of a four, five, or six cycles/session prescription, the first cycle was consistently responsible for the lowest proportion and the last cycle the greatest proportion of the net UF per session. This was not a last cycle effect because there was a significant difference between the UF volumes achieved among the first, middle, and last cycles. Cycle UFR and volumes gradually increased from the first to middle to last cycle.

Inadequate UF is a major cause of PD technique discontinuation. Thus, a better understanding of intercycle UF trends may inform prescription interventions that can improve patient retention. Our findings have several important current practice clinical implications. For example, clinicians should not judge an APD prescription's performance by the UF volumes achieved during the first few cycles. This is especially relevant in the setting of hospital APD where we advise postponing APD prescriptive changes because of UF issues until completion of at least four cycles. The same reasoning applies when assessing home APD treatment trends. A potential glucose-reducing solution to inadequate UF is the use of higher dextrose solutions early in an APD session, and lower dextrose solutions in later cycles, rather than increasing the dextrose concentration prescribed across all cycles. This might offset the low UF volumes seen in early cycles and reduce overall glucose exposure. The addition of another cycle to an existing prescription may be an option, as we have shown that UF volumes continue to increase as the cycles progress. We acknowledge that this will extend the treatment session, prolong glucose exposure, and increase solution utilization with its implications (cost, delivery, storage, *etc.*), but this would be a good option for patients with inadequate UF and no access to icodextrin.

There are several potential explanations for the observed increase in UF with subsequent cycles. There may be progressive recruitment of peritoneal membrane surface area because intraperitoneal fluid eventually finds pathways to areas inaccessible in earlier cycles. A similar but mechanistically different possibility is that mesenteric elasticity increases from one cycle to the next, resulting in the recruitment of more peritoneal surface area. Shear wave elastography has demonstrated that stiffer peritoneal membranes achieve lower UF.^11^ Another possible explanation is that there is a gradual increase in interstitial dextrose concentration as the cycles progress, resulting in greater osmotic pressures and subsequent increased intercycle UF volumes. While it might be argued that relatively elevated serum glucose concentrations at the initiation of APD treatments because of food ingestion (dinner) before treatments may have dampened the relative osmotic pressure gradient, we do not believe this to be an adequate explanation for our findings. Given that dialysate glucose content in PD dextrose solutions ranges from 1360 mg/dl (1.5% dextrose) to 3860 mg/dl (4.25%), it is highly unlikely—as per the Van't Hoff law of osmotic pressure—that even a state of relative postprandial hyperglycemia of 300 mg/dl would affect osmotic pressures enough to explain our findings. These possible explanations have clinical and physiologic implications.

Patients on tidal PD did not achieve significantly different night therapy or total UF volumes than their nontidal PD counterparts. When compared with patients without a last fill, those with a last fill achieved greater UF volumes in the first cycle and lower UF volumes in the last cycle (Supplemental Table 5). Our findings are similar to those of Stachowska-Pietka *et al.,*^10^ who found that patients who performed APD after a dry day (no last fill) had lower first cycle UF volumes compared with those who had a wet day (last fill). Using a spatially distributed model, they concluded that this was because of greater peritoneal tissue hydration in those with a last fill. We assume that tissue hydration refers to dextrose-rich water. Patients with a last fill may have relatively higher interstitial dextrose concentrations (osmotic pressures), facilitating water and solute transfer across the peritoneal membrane.^10^ In another study, Heimbürger *et al.*^12^ showed that maintaining a steady concentration of glucose in PD fluid—using the Carry Life UF device—over a single 5-hour dwell had higher UFR and sodium removal rate compared with a single 4-hour dwell of standard 2.5% dextrose. Combining the findings of both studies, along with ours, we believe that we are correct in interpreting peritoneal tissue hydration to be equivalent to interstitial dextrose concentration. As an APD treatment progresses from one cycle to the next, the interstitial dextrose concentration increases, thereby facilitating the increasing UF volumes we observed.

Davies^13^ suggests that peak glucose concentrations may be more membrane deleterious than steady state concentrations. One approach to a steady intraperitoneal dextrose (glucose) concentration is that of Heimbürger *et al.*,^12^ discussed above. However, another approach to steady and even lower interstitial glucose exposure is to apply continuous flow PD (CFPD). Models suggest that in CFPD, there would be greater UF than with conventional APD, with lower peak intraperitoneal glucose concentration and less daily glucose loading.^14–16^ Adequate UF can likely be achieved with lower glucose exposure in CFPD with a dextrose concentration of 0.5%. Further research into this application is warranted. Stagnation in the technology development is not justified.

Our study has several strengths. Treatment data were collected for seven consecutive PD treatment days, minimizing temporal alterations in peritoneal membrane characteristics. Each patient was on the same individual prescription, as prescribed by their provider during the seven days of data collection. All the patients, except for one, were using Baxter's Amia cycler, which insures the instilling of constant dextrose concentration solutions throughout the course of the session. Patients were followed for seven treatment days, and each individual treatment cycle data were used for analysis, rather than using the mean value for each cycle. All patients had their APD cycler temperatures set at the manufacturer's default of 36°C. There were also several limitations to the study. The sample size is relatively small. While dialysate temperature is an important factor in UF, we assume that the dialysate temperatures do not vary significantly among the cycles. Although patients' prescriptions and dialysate solutions used were verified with them and their providers, the Sharesource platform does not provide a safeguard by which we can ascertain that these were the solutions used. We also did not assess the effect of a prolonged drain time on outcomes or collect drain alarm data.

This is the first study investigating variations in UF volumes achieved between APD cycles. Given the increased usage of APD, it is important to understand the performance of APD cyclers. UF volumes achieved in APD differed from one cycle to the next. Adjusting for dextrose concentration, peritoneal transfer status, as well as linear, nonlinear, and devoid analyses of dwell time and fill volume, we demonstrated consistent cycle variability in UF volumes. As the APD treatment progressed, the UF achieved per cycle increased and so did the likelihood of net positive UF. While the exact explanation of these findings is unclear, we hypothesize that peritoneal surface area recruitment, mesenteric elasticity, and cumulative glucose concentration in the interstitium and over the course of an APD treatment are possible factors. Further investigation is needed to understand this physiology and especially as we seek an approach to minimizing total membrane glucose exposure.

## Supplementary Material

SUPPLEMENTARY MATERIAL

## Data Availability

Anonymized data created for the study are or will be available in a persistent repository upon publication. Recorded Data. Figshare. 10.6084/m9.figshare.26129287.

## Supplemental Material

Supplemental Table 1. Comparing total ultrafiltration and night therapy ultrafiltration volumes (ml) across different dextrose concentrations, transport statuses, and last fill.

Supplemental Table 2. Ratio of night therapy ultrafiltration to total ultrafiltration achieved per day.

Supplemental Table 3. Spearman's correlations between length of prescription and dwell times for each of the six PD cycles and total cycle time.

Supplemental Table 4. Comparing predicted cycle ultrafiltration between patients with a last fill and those without a last fill. Generalized estimating equation (GEE) nonlinear model analysis was used, and cycles were categorized into first, middle, and last. Adjustments were made for average dextrose concentration, dwell time, fill volume, transport status, and cycle number.

Supplemental Table 5. Predicting cycle ultrafiltration using the generalized estimating equation (GEE) nonlinear model analysis and recategorizing cycles into first, middle, and last. Adjustments were made for average dextrose concentration, transfer status, and cycle number, and without adjusting for dwell time or fill volume.
