# Supplementary material for: Ultrafiltration Patterns during Automated Peritoneal Dialysis: Findings and Insights to Peritoneal Physiology
Source: Kidney360. 2024 Jul 8;5(11):1683–91. doi: 10.34067/KID.0000000000000506 (PMC12282617; doi:10.34067/KID.0000000000000506)
Supplement: SUPPLEMENTARY MATERIAL [file kidney360-5-1683-s001.pdf]

ASN American Society of Nephrology | JASN Journal of the American Society of Nephrology | KIDNEY360 Accessing Our World From Every Angle | CJASN Clinical Journal of the American Society of Nephrology

ASN Journal Disclosure Form

As per ASN journal policy, I have disclosed any financial relationships or commitments I have held in the past 36 months as included below. I have listed my Current Employer below to indicate there is a relationship requiring disclosure. If no relationship exists, my Current Employer is not listed.

N. Abudaff reports the following:
Employer: Arizona Kidney Disease & Hypertension Centers

I understand that the information above will be published within the journal article, if accepted, and that failure to comply and/or to accurately and completely report the potential financial conflicts of interest could lead to the following: 1) Prior to publication, article rejection, or 2) Post-publication, sanctions ranging from, but not limited to, issuing a correction, reporting the inaccurate information to the authors' institution, banning authors from submitting work to ASN journals for varying lengths of time, and/or retraction of the published work.

Name: Naief N. Abudaff
Manuscript ID: K360-2024-000248R1
Manuscript Title: Ultrafiltration Patterns during Automated Peritoneal Dialysis: Findings and Insights to Peritoneal Physiology,
Date of Completion: June 4, 2024
Disclosure Updated Date: May 6, 2024

ASN
American Society of Nephrology
JASN
Journal of the American Society of Nephrology
KIDNEY360
Accessing Our World From Every Angle
CJASN
Clinical Journal of the American Society of Nephrology

ASN Journal Disclosure Form

As per ASN journal policy, I have disclosed any financial relationships or commitments I have held in the past 36 months as included below. I have listed my Current Employer below to indicate there is a relationship requiring disclosure. If no relationship exists, my Current Employer is not listed.

J. Arroyo Ornelas reports the following:
Employer: Vanderbilt University Medical Center

I understand that the information above will be published within the journal article, if accepted, and that failure to comply and/or to accurately and completely report the potential financial conflicts of interest could lead to the following: 1) Prior to publication, article rejection, or 2) Post-publication, sanctions ranging from, but not limited to, issuing a correction, reporting the inaccurate information to the authors' institution, banning authors from submitting work to ASN journals for varying lengths of time, and/or retraction of the published work.

Name: Juan Pablo Arroyo Ornelas
Manuscript ID: K360-2024-000248R1
Manuscript Title: Ultrafiltration Patterns during Automated Peritoneal Dialysis: Findings and Insights to Peritoneal Physiology
Date of Completion: June 11, 2024
Disclosure Updated Date: May 7, 2024

ASN American Society of Nephrology | JASN Journal of the American Society of Nephrology | KIDNEY360 Accessing Our World From Every Angle | CJASN Clinical Journal of the American Society of Nephrology

ASN Journal Disclosure Form

As per ASN journal policy, I have disclosed any financial relationships or commitments I have held in the past 36 months as included below. I have listed my Current Employer below to indicate there is a relationship requiring disclosure. If no relationship exists, my Current Employer is not listed.

O. El Shamy reports the following:
Employer: George Washington University; Consultancy: Outset Medical; Honoraria: Home Dialysis University ; UpToDate; and Advisory or Leadership Role: Light Line Medical.

I understand that the information above will be published within the journal article, if accepted, and that failure to comply and/or to accurately and completely report the potential financial conflicts of interest could lead to the following: 1) Prior to publication, article rejection, or 2) Post-publication, sanctions ranging from, but not limited to, issuing a correction, reporting the inaccurate information to the authors' institution, banning authors from submitting work to ASN journals for varying lengths of time, and/or retraction of the published work.

Name: Osama El Shamy
Manuscript ID: K360-2024-000248R1
Manuscript Title: Ultrafiltration Patterns during Automated Peritoneal Dialysis: Findings and Insights to Peritoneal Physiology
Date of Completion: June 4, 2024
Disclosure Updated Date: May 21, 2024

ASN American Society of Nephrology · JASN Journal of the American Society of Nephrology · KIDNEY360 Accessing Our World From Every Angle · CJASN Clinical Journal of the American Society of Nephrology

ASN Journal Disclosure Form

As per ASN journal policy, I have disclosed any financial relationships or commitments I have held in the past 36 months as included below. I have listed my Current Employer below to indicate there is a relationship requiring disclosure. If no relationship exists, my Current Employer is not listed.

T. Golper reports the following:
Consultancy: NxStage; Lightline; Research Funding: Renal Research Institute; Honoraria: Up To Date; NxStage; Renal Research Institute, , Home Dialysis University; Patents or Royalties: Up to Date; Advisory or Leadership Role: NxStage; LightLine Medical,: Home Dialysis University; and Other Interests or Relationships: Section Editor for Dialysis Up To Date; Executive Committee Home Dialysis University.

I understand that the information above will be published within the journal article, if accepted, and that failure to comply and/or to accurately and completely report the potential financial conflicts of interest could lead to the following: 1) Prior to publication, article rejection, or 2) Post-publication, sanctions ranging from, but not limited to, issuing a correction, reporting the inaccurate information to the authors' institution, banning authors from submitting work to ASN journals for varying lengths of time, and/or retraction of the published work.

Name: Thomas A. Golper
Manuscript ID: K360-2024-000248R1
Manuscript Title: Ultrafiltration Patterns during Automated Peritoneal Dialysis: Findings and Insights to Peritoneal Physiology
Date of Completion: June 4, 2024
Disclosure Updated Date: May 6, 2024

ASN American Society of Nephrology | JASN Journal of the American Society of Nephrology | KIDNEY360 Accessing Our World From Every Angle | CJASN Clinical Journal of the American Society of Nephrology

ASN Journal Disclosure Form

As per ASN journal policy, I have disclosed any financial relationships or commitments I have held in the past 36 months as included below. I have listed my Current Employer below to indicate there is a relationship requiring disclosure. If no relationship exists, my Current Employer is not listed.

R. Greevy reports the following:
Employer: Vanderbilt University Medical Center

I understand that the information above will be published within the journal article, if accepted, and that failure to comply and/or to accurately and completely report the potential financial conflicts of interest could lead to the following: 1) Prior to publication, article rejection, or 2) Post-publication, sanctions ranging from, but not limited to, issuing a correction, reporting the inaccurate information to the authors' institution, banning authors from submitting work to ASN journals for varying lengths of time, and/or retraction of the published work.

Name: Robert Greevy
Manuscript ID: K360-2024-000248R1
Manuscript Title: Ultrafiltration Patterns during Automated Peritoneal Dialysis: Findings and Insights to Peritoneal Physiology
Date of Completion: June 14, 2024
Disclosure Updated Date: May 7, 2024

ASN
American Society of Nephrology
JASN
Journal of the American Society of Nephrology
KIDNEY360
Accessing Our World From Every Angle
CJASN
Clinical Journal of the American Society of Nephrology

ASN Journal Disclosure Form

As per ASN journal policy, I have disclosed any financial relationships or commitments I have held in the past 36 months as included below. I have listed my Current Employer below to indicate there is a relationship requiring disclosure. If no relationship exists, my Current Employer is not listed.

A. Guide reports the following:
Employer: Vanderbilt University Medical Center

I understand that the information above will be published within the journal article, if accepted, and that failure to comply and/or to accurately and completely report the potential financial conflicts of interest could lead to the following: 1) Prior to publication, article rejection, or 2) Post-publication, sanctions ranging from, but not limited to, issuing a correction, reporting the inaccurate information to the authors' institution, banning authors from submitting work to ASN journals for varying lengths of time, and/or retraction of the published work.

Name: Andrew Guide
Manuscript ID: K360-2024-000248R1
Manuscript Title: Ultrafiltration Patterns during Automated Peritoneal Dialysis: Findings and Insights to Peritoneal Physiology
Date of Completion: June 10, 2024
Disclosure Updated Date: June 10, 2024

ASN
American Society of Nephrology
JASN
Journal of the American Society of Nephrology
KIDNEY360
Accessing Our World From Every Angle
CJASN
Clinical Journal of the American Society of Nephrology

ASN Journal Disclosure Form

As per ASN journal policy, I have disclosed any financial relationships or commitments I have held in the past 36 months as included below. I have listed my Current Employer below to indicate there is a relationship requiring disclosure. If no relationship exists, my Current Employer is not listed.

S. Patel reports the following:
Employer: Renal Associates of Columbus, LLC

I understand that the information above will be published within the journal article, if accepted, and that failure to comply and/or to accurately and completely report the potential financial conflicts of interest could lead to the following: 1) Prior to publication, article rejection, or 2) Post-publication, sanctions ranging from, but not limited to, issuing a correction, reporting the inaccurate information to the authors' institution, banning authors from submitting work to ASN journals for varying lengths of time, and/or retraction of the published work.

Name: Sagar Patel
Manuscript ID: K360-2024-000248R1
Manuscript Title: Ultrafiltration Patterns during Automated Peritoneal Dialysis: Findings and Insights to Peritoneal Physiology,
Date of Completion: June 4, 2024
Disclosure Updated Date: May 6, 2024

ASN American Society of Nephrology | JASN Journal of the American Society of Nephrology | KIDNEY360 Accessing Our World From Every Angle | CJASN Clinical Journal of the American Society of Nephrology

ASN Journal Disclosure Form

As per ASN journal policy, I have disclosed any financial relationships or commitments I have held in the past 36 months as included below. I have listed my Current Employer below to indicate there is a relationship requiring disclosure. If no relationship exists, my Current Employer is not listed.

A. Shah reports the following:
Employer: Brown Physicians Inc; Consultancy: Otsuka, Calliditas, CareDX; Research Funding: Otsuka; Honoraria: JSOM, USC-Greenville, CTC-RI, NKF-CRNNE, ARA/IRC, Greenfield; and Advisory or Leadership Role: American College of Physicians Rhode Island Chapter Governors Advisory Council ; American Society of Nephrology Policy and Advocacy Committee; Renal Physicians Association Government Affairs Committee; Renal Physicians Association Policy Advocacy Leadership Steering Committee.

I understand that the information above will be published within the journal article, if accepted, and that failure to comply and/or to accurately and completely report the potential financial conflicts of interest could lead to the following: 1) Prior to publication, article rejection, or 2) Post-publication, sanctions ranging from, but not limited to, issuing a correction, reporting the inaccurate information to the authors' institution, banning authors from submitting work to ASN journals for varying lengths of time, and/or retraction of the published work.

Name: Ankur Shah
Manuscript ID: K360-2024-000248R1
Manuscript Title: "Ultrafiltration Patterns during Automated Peritoneal Dialysis: Findings and Insights to Peritoneal Physiology
Date of Completion: May 3, 2024
Disclosure Updated Date: May 2, 2024

ASN American Society of Nephrology | JASN Journal of the American Society of Nephrology | KIDNEY360 Accessing Our World From Every Angle | CJASN Clinical Journal of the American Society of Nephrology

ASN Journal Disclosure Form

As per ASN journal policy, I have disclosed any financial relationships or commitments I have held in the past 36 months as included below. I have listed my Current Employer below to indicate there is a relationship requiring disclosure. If no relationship exists, my Current Employer is not listed.

N. Wyatt has nothing to disclose.

I understand that the information above will be published within the journal article, if accepted, and that failure to comply and/or to accurately and completely report the potential financial conflicts of interest could lead to the following: 1) Prior to publication, article rejection, or 2) Post-publication, sanctions ranging from, but not limited to, issuing a correction, reporting the inaccurate information to the authors' institution, banning authors from submitting work to ASN journals for varying lengths of time, and/or retraction of the published work.

Name: Nicole Wyatt
Manuscript ID: K360-2024-000248R1
Manuscript Title: Ultrafiltration Patterns during Automated Peritoneal Dialysis: Findings and Insights to Peritoneal Physiology
Date of Completion: June 4, 2024
Disclosure Updated Date: May 6, 2024
