# Supplementary material for: Ultrafiltration Patterns during Automated Peritoneal Dialysis: Findings and Insights to Peritoneal Physiology
Source: Kidney360. 2024 Jul 8;5(11):1683–91. doi: 10.34067/KID.0000000000000506 (PMC12282617; doi:10.34067/KID.0000000000000506)
Supplement: SUPPLEMENTARY MATERIAL [file kidney360-5-1683-s002.pdf]

Supplemental Materials

**Table S1**	Comparing total ultrafiltration and night therapy ultrafiltration volumes (ml) across different dextrose concentrations, transport statuses and last fill
**Table S2**	Ratio of night therapy ultrafiltration to the total ultrafiltration achieved per day
**Table S3**	Spearman's correlations between length of prescription and dwell times for each of the six PD cycles, and total cycle time
**Table S4**	Comparing predicted cycle ultrafiltration between patients with a last fill and those without a last fill. Generalized Estimating Equations (GEE) non-linear model analysis was used, and cycles were categorized into First, Middle, and Last. Adjustments were made for average dextrose concentration, dwell time, fill volume, transport status, and cycle number
**Table S5**	Predicting cycle ultrafiltration using the Generalized Estimating Equations (GEE) non-linear model analysis, and recategorizing cycles into First, Middle, and Last. Adjustments were made for average dextrose concentration, transfer status, and cycle number, and without adjusting for dwell time or fill volume

Table S1. Comparing total ultrafiltration and night therapy ultrafiltration volumes (ml) across different dextrose concentrations, transport statuses and last fill

Variable	Total Ultrafiltration	Night Therapy Ultrafiltration
**Average Dextrose Concentration**		
1.5	578 (37-1,214)	396 (206-1,213)
2.0 (Mix 1.5 & 2.5)	750 (453-1,017)	666 (395-1,034)
2.5	1,128 (815-1,405)	1,076 (698-1,309)
3.4 (Mix 2.5 & 4.25)	1,425 (1,145-1,758)	1,485 (1,039-1,643)
**Transfer Status**		
High	1,250 (622-1,717)	655 (318-1,267)
High Average	889 (611-1,236)	779 (478-1,150)
Low Average	999 (669-1,358)	1,096 (716-1,412)
Low	1,412 (1,269-1,526)	1,159 (1,082-1,223)
**Last Fill**		
Yes	1,065 (753-1,427)	948 (493-1,293)
No	868 (563-1,181)	1,019 (611-1,215)

Table S2. Ratio of night therapy ultrafiltration to the total ultrafiltration achieved per day

	**Cycle 1**	**Cycle 2**	**Cycle 3**	**Cycle 4**	**Cycle 5**	**Cycle 6**
4 Cycles	-0.1<br>(-0.3 – 0.1)	0.2<br>(0.1 – 0.4)	0.2<br>(0.0 – 0.4)	0.6<br>(0.1 – 1.1)	N/A	N/A
5 Cycles	0.0<br>(-0.2 – 0.2)	0.2<br>(0.0 – 0.3)	0.2<br>(0.0 – 0.4)	0.1<br>(0.0 – 0.3)	0.3<br>(0.1 – 0.7)	N/A
6 Cycles	0.1<br>(0.0 – 0.2)	0.1<br>(0.0 – 0.3)	0.1<br>(0.1 – 0.3)	0.1<br>(0.0 – 0.2)	0.1<br>(0.1 – 0.2)	0.2<br>(0.1 – 0.4)
Ultrafiltration Ratio (All Patients)	0.0<br>(-0.2 – 0.2)	0.1<br>(0.0 – 0.3)	0.2<br>(0.0 – 0.4)	0.2<br>(0.0 – 0.5)	0.2<br>(0.1 – 0.6)	0.2<br>(0.1 – 0.4)

Table S3. Spearman's correlations between length of prescription and dwell times for each of the six PD cycles, and total cycle time*

Variable 1	Variable 2	Correlation (95% CI)
Prescribed length of PD treatment	Total Cycle Time	0.86 (0.82, 0.89)
	**Dwell Time**	
	Cycle 1	0.45 (0.22, 0.64)
	Cycle 2	0.40 (0.16, 0.60)
	Cycle 3	0.36 (0.11, 0.56)
	Cycle 4	0.44 (0.20, 0.63)
	Cycle 5	0.38 (0.14, 0.58)
	Cycle 6	0.44 (0.20, 0.62)

*Cycle time = fill time + dwell time + drain time

Table S4. Comparing predicted cycle ultrafiltration between patients with a last fill and those without a last fill. Generalized Estimating Equations (GEE) non-linear model analysis was used, and cycles were categorized into First, Middle, and Last. Adjustments were made for average dextrose concentration, dwell time, fill volume, transport status, and cycle number*

**Variable**	**Estimate (Std Error)**	
	**Last Fill**	**No Last Fill**
**Average Dextrose Concentration**		
1.5%	-40.79 (69.92)	7.93 (83.93)
2.5%	47.76 (29.06)	148.82 (29.53)**
3.4% (Mix 2.5 & 4.25)	52.89 (37.15)	-
**Dwell Time**	-31.30 (40.91)	-134.00 (91.62)
**Fill Volume**	17.60 (552.58)	108.00 (119.51)
**Transfer Status**		
High Average	-5.66 (20.40)	-
Low Average	163.27 (24.58)**	2.43 (34.79)
Low	81.24 (27.02)**	-
Unavailable	-28.53 (16.14)	-0.18 (31.92)
**Cycle**		
First	-171.15 (43.43)**	-334.19 (49.01)**
Last	257.35 (86.93)**	315.78 (96.36)**

*Reference categories were: Average Dextrose Concentration 2.0, Transfer Status: High, and Middle Cycle.

**p-value < 0.05

Table S5. Predicting cycle ultrafiltration using the Generalized Estimating Equations (GEE) non-linear model analysis, and recategorizing cycles into First, Middle, and Last. Adjustments were made for average dextrose concentration, transfer status, and cycle number, and without adjusting for dwell time or fill volume*

Variable	Estimate (Std Error)	p-value
**Average Dextrose Concentration**		
1.5%	29.21 (65.57)	0.656
2.5%	70.49 (29.79)	0.018
3.4% (Mix 2.5 & 4.25)	135.44 (27.49)	< 0.001
**Transfer Status**		
High Average	17.84 (31.31)	0.569
Low Average	94.43 (35.74)	0.008
Low	59.20 (42.81)	0.167
Unavailable	-20.62 (36.41)	0.571
**Cycle**		
First	-221.28 (34.81)	< 0.001
Last	273.97 (66.13)	< 0.001

*Reference categories were: Average Dextrose Concentration 2.0, Transfer Status: High, and Middle Cycle.
